# Mystery of gamma wave stimulation in brain disorders

**DOI:** 10.1186/s13024-024-00785-x

**Published:** 2024-12-18

**Authors:** Qianting Deng, Chongyun Wu, Emily Parker, Jing Zhu, Timon Cheng-Yi Liu, Rui Duan, Luodan Yang

**Affiliations:** 1 School of Physical Education and Sports Science, South China Normal University, Guangzhou, 510006 China; 2 Department of Respiratory and Critical Care Medicine, The Central Hospital of Wuhan, Tongji Medical College, Huazhong University of Science and Technology, Wuhan, 430000 China; 3 Augusta University, 1120 15th Street, Augusta, GA 30912 USA

**Keywords:** Gamma entrainment, γ oscillations, Brain stimulation, Memory, Neurological function, Alzheimer’s disease

## Abstract

Neuronal oscillations refer to rhythmic and periodic fluctuations of electrical activity in the central nervous system that arise from the cellular properties of diverse neuronal populations and their interactions. Specifically, gamma oscillations play a crucial role in governing the connectivity between distinct brain regions, which are essential in perception, motor control, memory, and emotions. In this context, we recapitulate various current stimulation methods to induce gamma entrainment. These methods include sensory stimulation, optogenetic modulation, photobiomodulation, and transcranial electrical or magnetic stimulation. Simultaneously, we explore the association between abnormal gamma oscillations and central nervous system disorders such as Alzheimer’s disease, Parkinson’s disease, stroke, schizophrenia, and autism spectrum disorders. Evidence suggests that gamma entrainment-inducing stimulation methods offer notable neuroprotection, although somewhat controversial. This review comprehensively discusses the functional role of gamma oscillations in higher-order brain activities from both physiological and pathological perspectives, emphasizing gamma entrainment as a potential therapeutic approach for neuropsychiatric disorders. Additionally, we discuss future opportunities and challenges in implementing such strategies.

## Introduction

**Fig. 1:**
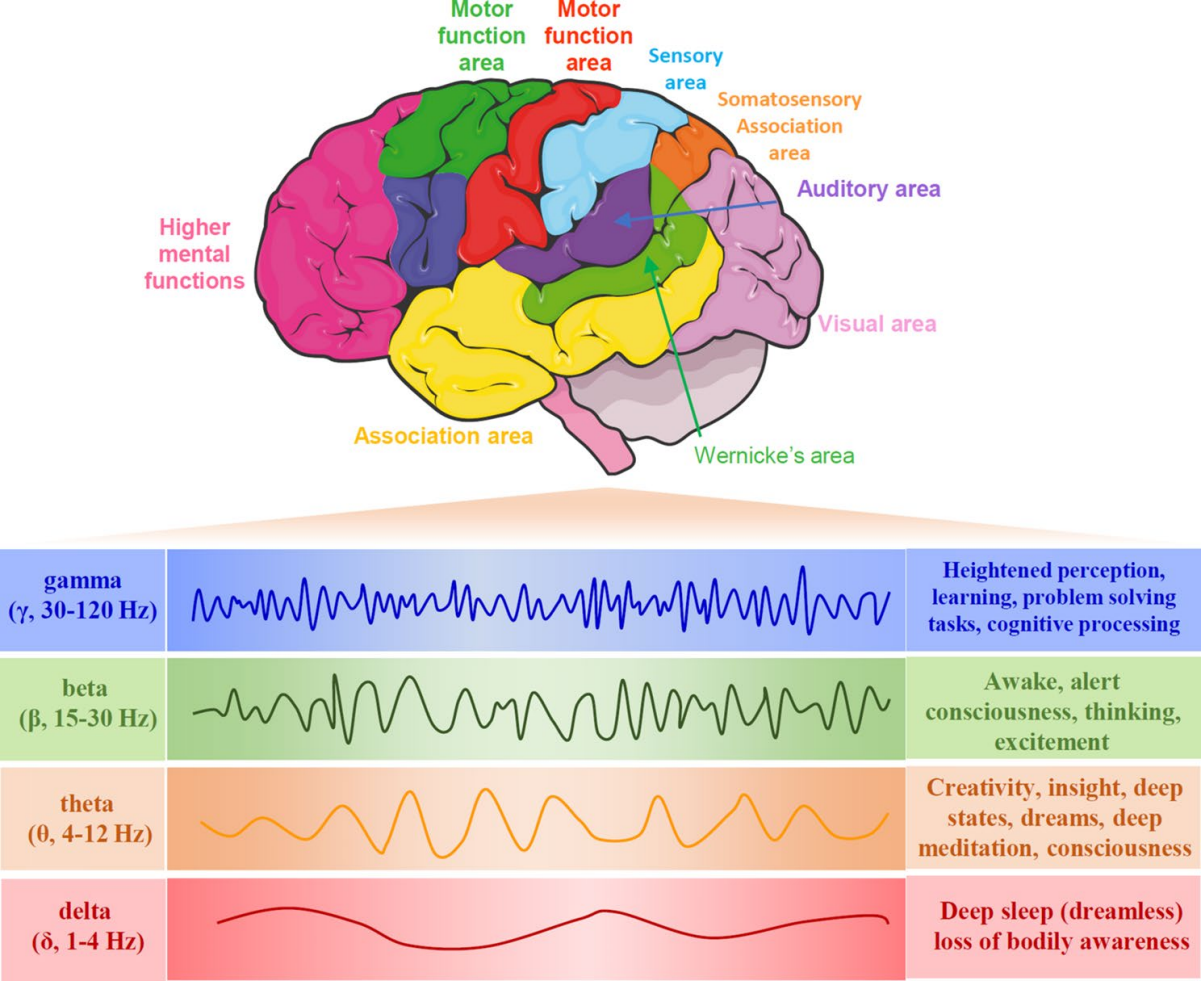
Diagram illustrating brain oscillation at different frequencies, including delta (δ, 1–4 Hz), theta (θ, 4–12 Hz), beta (β, 15–30), and gamma (γ, 30–80 Hz) oscillations. The γ oscillation is associated with heightened perception, learning, problem-solving tasks, and cognitive processing

Brain oscillations refer to rhythmic brain activity [1]. Endogenous brain oscillations occur at different frequencies, including delta (δ, 1–4 Hz), theta (θ, 4–12 Hz), beta (β, 15–30), and gamma (γ, 30–80 Hz) bands [2, 3] (Fig. 1). Additionally, alterations in oscillatory power are observed across a broad frequency range (80–250 Hz), known as the high-gamma band [4]. Gamma rhythms in various brain regions are believed to be integral to information storing and processing [5]. In the hippocampal CA1 region, these frequency bands specifically manifest during distinct phases of hippocampal coding, suggesting that they facilitate the routing of information originating from various brain areas to CA1 [6]. Especially, the low γ rhythms emanating from the primary visual cortex tend to process higher spatial frequency information [7]. Moreover, γ oscillations have been extensively investigated in the cortex, hippocampus, amygdala, olfactory bulb, striatum, and brainstem and found to play a critical role in sensory processing [8], perceptual integration [9], recognition, working memory [10], locomotion [11], and emotion [12]. In contrast, disrupted γ oscillations induce aberrant neural activity and brain dysfunction (Table 1) [13]. For example, disrupted γ oscillations cause dysregulation of neural circuits involved in cognitive function, exacerbating Alzheimer’s disease (AD) pathology [14, 15]. Furthermore, in an animal model of depression-like behaviors, including Flinders sensitive line (FSL) rats and mice expressing the truncated Disrupted-in-schizophrenia 1 (Disc1) mutation, γ oscillation abnormalities are observed [16, 17]. Emerging evidence suggests that the abnormal γ oscillations could be a biomarker for major depression [18].

**Table 1:** Aberrant γ oscillations in central nervous system diseases

Subject	Pathophysiology	Neural oscillations	Characteristics	Ref.
**Alzheimer’s disease**				
TgF344-AD rats	Synaptic dysfunction Neuronal hyperexcitability	SWR power ↑ SWR duration ↓ θ-γ coupling↓	Cognitive impairment	[139]
C57BL/6, PV-Cre knock-in mice, SST-IRES-Cre knock-in mice	Aβo1-42 causes synapse-specific dysfunctions in PV and SST interneurons	θ-nested γ oscillations↓ LTP↓	Memory encoding dysfunction	[140, 143]
(APP)/PS1 mice	Dysfunction of reciprocal dendrodendritic synapses between GCs and MCs	LFP↑ Aberrant increase in γ oscillations↑	Olfactory impairment preceding learning defect	[152]
APP/PS1 and 3xTg mice	Decrease in the excitatory responses of M/T cells	Ability of M/T cells to trigger interneuron GABA release↓	Olfactory dysfunction	[153]
Human apoE4-KI C57BL/6 mice	ApoE4-induced GABAergic interneuron loss	SWR-associated slow gamma power in the hippocampus↓	Learning and memory deficits	(14, 207)
C57BL/6 mice	Loss of tau homeostasis in hilar astrocytes of the dentate gyrus; Altered mitochondrial dynamics and function	Gamma oscillations and the number of neurons expressing PV in the dentate gyrus↓	Spatial memory impairments	[150]
**Parkinson’s disease**				
PD patients	Dopamine depletion The basal ganglia function disruption	Recruitment of fast gamma bursts during movement ↓	Bradykinesia	[169]
PD patients	LTP-like plasticity capacity in M1↓	γ oscillations within the basal ganglia-thalamocortical network↓	Locomotor dysfunction	[123]
C57BL/6 mice	Dopamine depletion selectively disrupts interactions between striatal neuron subtypes and LFP oscillations	Striatal transient high-γ (60–100 Hz) power ↑	Movement initiation and rotation impairment	(208)
**Stroke**				
C57/BL6J mice	Enduring depolarization and interneuron function impairment	The activity of adjacent excitatory neurons↓	Vascular and behavioral dysfunction	[29]
Two-vessel occlusion (2VO) rat model	Reduction of the theta-gamma cross-frequency coupling strength in the hippocampus	Short and long-term potentiation impairment	Cognitive dysfunction	[179]
**Schizophrenia**				
Sdy mice	Dysbindin-1 mutation-induced defective mitochondrial fission	Gamma range integrated power in CA3 ↓	Cognitive impairment	[186]
Dlx5/6(+/-) mice	Abnormalities in GABAergic interneurons	FSINs generate gamma oscillations↓	Disrupt PFC-dependent cognition	[68]
**Autism spectrum disorder**				
ASD patients	The number of interneurons↓ Dysregulation in GABA receptor subunit expression	Imbalance between excitatory and inhibitory signaling	Impairments in activities of daily living	[37]
ASD patients	Spontaneous gamma activity in frontal, temporal, and right-lateral regions↓	Task-related gamma power↓; Long- and short-range gamma connectivity↓	Sensory abnormalities	[190]

SWR, sharp wave-ripple; PV, parvalbumin interneurons; SST, somatostatin interneurons; LTP, long-term potentiation; GCs, Granule cells; MCs, mitral cells; LFP, local field potential; OSNs, olfactory sensory neurons; EOG, electro-olfactogram; M/T cells, mitral/tufted cells; M1, primary motor cortex; FSINs, fast-spiking interneurons; PFC, prefrontal cortex

Multiple studies demonstrate the beneficial effects of γ oscillation stimulation (Table 2) [19–22]. Currently, γ stimulations are conducted using a variety of methods, including non-invasive techniques such as sound [23], light [24], electricity [25], and magnetism [26], as well as invasive methods like optogenetic stimulation [27]. Promisingly, γ stimulation produced by non-invasive or invasive approaches has been shown to exert potent neuroprotective effects in brain disorders [24, 28, 29]. Parvalbumin-expressing (PV+) interneurons, which innervate the perisomatic regions of pyramidal neurons, are believed to be pivotal in regulating and sustaining γ oscillations within the brain [30]. Substantial evidence supports the notion that modulating γ oscillations affects neurocircuit function and behavior [11, 20, 31, 32]. Therefore, this review provides an overview of current research progress on the potential therapeutic effects of γ oscillations in various brain disorders. Furthermore, this review focuses on moderating γ activity in the brain through external stimulation, particularly on 40 Hz γ activity.

**Table 2:** The effect of gamma entrainment in central nervous system diseases

Method	Protocol		Subject	Outcome	Behavior	Ref.	
**Cognitive disorders**							
Optogenetic activation of PV and SST interneurons	5 Hz		C57BL/6 mice, PV-Cre knock-in mice, SST-IRES-Cre knock-in mice	Restores theta-nested gamma oscillations and oscillation-induced spike timing-dependent LTP	Memory encoding↑ The execution of cognitive function↑	[140]	
Auditory or audiovisual stimulation	40 Hz, 2 h/day, 14 days		ApoE4 Knock-In Mice	Amyloid protein levels↓ Neuronal apoptosis↓ cholinergic transmission↑	Cognitive performance↑ Neuropathology↓	[158]	
Optogenetic stimulation of parvalbumin neurons	40 Hz		J20-APP AD mouse 5XFAD mice	Slow gamma oscillations amplitude and phase-amplitude coupling↑ Aβ deposition↑	Spatial memory↑	[2, 106]	
Optogenetic stimulation of FS-PV interneurons	40 Hz, 1 h/day		5XFAD mice	Aβ1–40 and Aβ1–42 isoforms level↓ Microglial Aβ uptake ↑	Cognitive function↑	[48]	
Visual stimulation	40 Hz, 1 h/day, 7 days		5XFAD mice	Aβ levels↓ Microglial Aβ uptake↑	Cognitive function↑	[48]	
Chronic daily gamma visual entrainment	40 Hz, 1 h/day, 22 days		Tau P301S mice CK-p25 mice	Neuronal loss↓ DNA damage↓ Synaptic function ↑ Neuroprotective factors↑	Learning and spatial memory ↑ Neurodegeneration↓	[21]	
Combined visual and auditory stimulation	40 Hz, 1 h/d, for 7 days		5XFAD mice	Aβ levels↓ Tau phosphorylation↓ Reactive astrocytes and microglia↑	Recognition and spatial memory↑	[20]	
Transcranial focused ultrasound	40 Hz		5XFAD mice	Microglia activation↑ Aβ plaque clearance↑	Learning and memory↑		[159]
Transcranial alternating current stimulation	40 Hz, 1 h/day, 4 weeks		Patients with mild-to-moderate dementia (AD)	p-Tau burden temporal lobe regions↓	Cognitive function↑	[166]	
Visual stimulation	30–50 Hz, 1 h/day, 14 days		two-vessel occlusion (2VO) rat model	Reinstated the synchronization of phase-amplitude coupling with theta oscillations	Degeneration↓ Cognitive function↑	[49]	
**Mental disorders**							
Visual stimulation	40 Hz, 1 h/day, 30 days		APP/PS1 AD mouse	Aβ deposition↓ Clock proteins expression↑	Circadian rhythm disorders↓	[22]	
Chronic multisensory gamma stimulation	40 Hz, 20 min per session, 3 sessions per block		C57BL/6 PD mice	p-α-Syn deposition↓ Stress-related ACTH and corticosterone levels↓	Depressive behaviors↓	[173]	
Visual stimulation	40 Hz, 2 h/d, for 21 days		C57BL/6 stroke mice	Anxiety susceptibility to stress exposure ↓ Microglia activation ↓	anxiety-like behaviors↓	[78]	
		Motor disorders					
iTBS-γ tACS costimulation	70 Hz (γ-tACS) and 20 Hz (β-tACS)		PD patients	LTP-like plasticity↑ Facilitation of MEPs↑	Motor function↑	[123]	
Sensory stimulation	40 Hz, 2 h/day, 1 month		C57BL/6 PD mice	α-Syn clearance↑ Cell apoptosis in M1↓	Neuromuscular strength↑	[173]	
Vibration at gamma frequency	40 Hz, 25 min/day, 12 weeks		PD patients	Tremor↓ Rigidity↓ Bradykinesia↓	Motor symptoms↑	[174]	
Deep brain stimulation	160 Hz		PD patients	The cross-frequency interactions between finely tuned gamma oscillations↑	Motor performance ↑ Beta power ↓ Gamma power ↑	(209)	
Optogenetic stimulation of interneurons	40 Hz, 1 h/d		C57/BL6J stroke mice	Spreading depolarizations Cerebral blood flow ↑	Motor performance ↑ Brain swelling and lesion volume ↓	[29]	
Optogenetic stimulation of the nucleus basalis	20 Hz		Thirty-five adult ChAT-Cre/Ai32(ChR2-YFP)	Acetylcholine↑ Improved recovery of reaching and movement scores	Functional recovery↑ Motor behavior↑	[176]	

PV, parvalbumin interneurons; SST, somatostatin interneurons; LTP, long-term potentiation; FS-PV, fast-spiking, parvalbumin-positive interneurons; VC, visual cortex; AC, auditory cortex; mPFC, medial prefrontal cortex; ACTH, adreno-cortico-tropic-hormone; MEPs, motor-evoked potentials;

### Gamma oscillations

Gamma oscillations are rhythmic fluctuations across multiple brain regions, characterized by local field potential changes and interareal coherence, and aid in sensory information processing, attentional selection, and memory operations [33, 34]. For example, enhanced γ activity is observed in the neocortex and hippocampus during sensory information transmission and in interareal coherence [34]. As mentioned above, gamma rhythms are categorized into narrowband gamma (i.e., gamma oscillations) and broadband γ (i.e., high gamma), which exert different biophysical effects [35]. Whereas narrowband gamma represents a “true” gamma oscillation, broadband gamma often represents a non-oscillatory or “aperiodic” electroencephalography (EEG) phenomenon [35]. Mechanistically, the emergence of γ oscillations has been attributed to γ-aminobutyric acid type A (GABAA) receptor-mediated inhibition involving interactions between fast-spiking and PV + interneurons [36, 37]. Furthermore, functional differences in PV + interneurons are observed in multiple disorders, disrupting the excitation/inhibition balance and causing abnormalities in γ oscillations [38, 39]. Empirical evidence has elucidated that optogenetic stimulation of PV + interneurons amplifies oscillatory γ activity, while inhibition of PV + interneurons diminishes γ oscillations [40]. For instance, therapeutics designed to target PV + interneurons specifically have been shown to restore normal γ oscillation patterns, thereby enhancing the cognitive function of the J20-APP AD mouse model through optogenetic interventions [2].

With evidence highlighting the critical role of γ oscillations in sensory and cognitive processes, researchers have investigated the presence of abnormal γ oscillations in neurological and neuropsychiatric conditions [41, 42]. Indeed, γ-frequency oscillations are disrupted in various brain disorders, including AD [14], Parkinson’s disease (PD) [43], stroke [44], Schizophrenia (SCZ) [45], and autism spectrum disorder (ASD) [46]. These disrupted γ-frequency oscillations impair neuronal encoding and sensory and/or cognitive information transformation [30, 35]. Research suggests that γ oscillations may serve as potential biomarkers for neural imbalances or interneuron dysfunction, reflecting the underlying pathophysiological mechanisms of essential neural functions in neuropsychiatric diseases [40, 47, 48]. Thus, reinstating normal γ activity is a potential therapy for improving higher-order cognition, sensory-motor integration, working memory, attention, perceptual binding, and network synchronization.

## Sensory stimulation methods to induce gamma oscillations

**Fig. 2:**
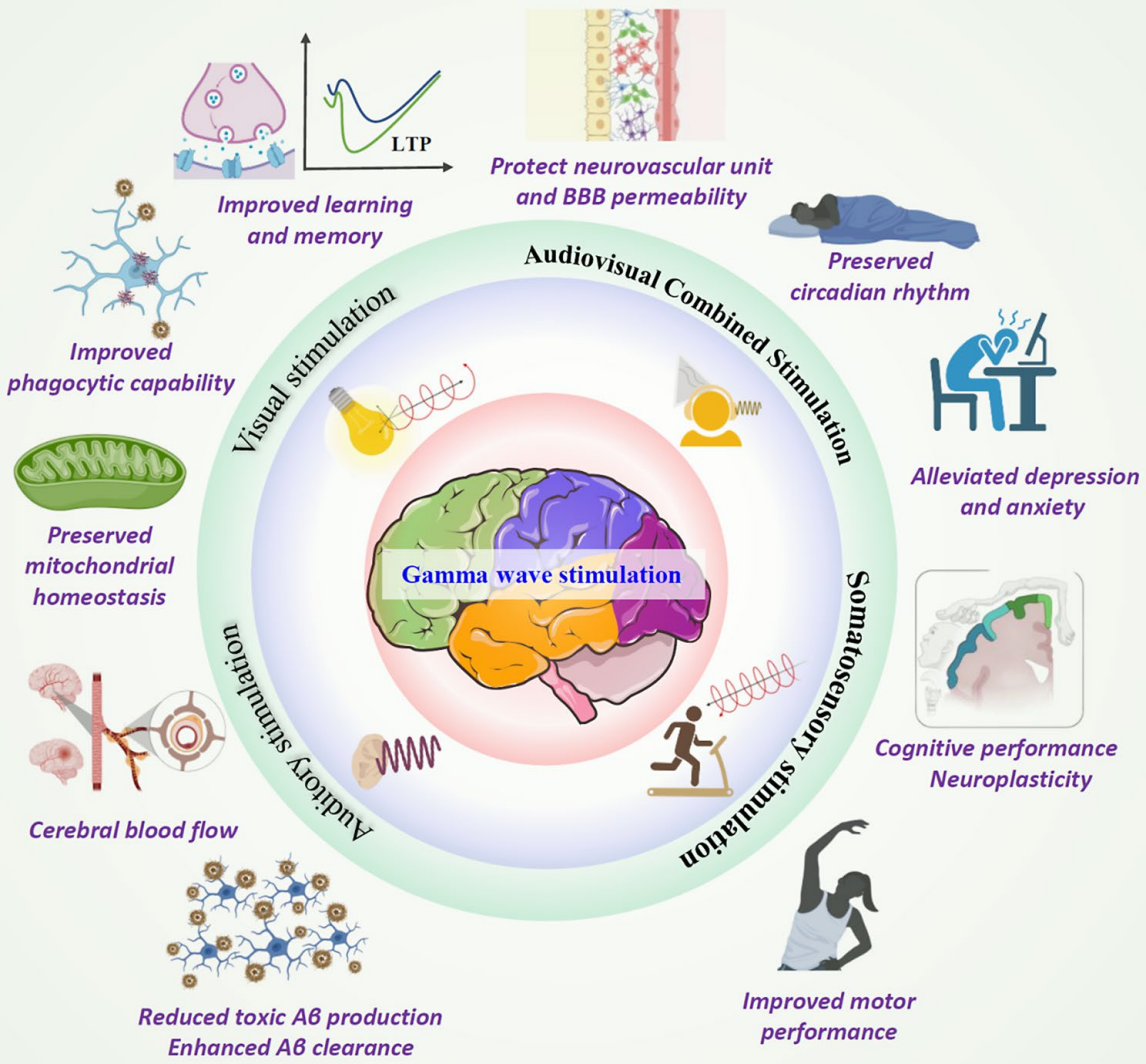
Gamma entrainment using sensory stimuli (GENUS). GENUS encompasses a range of methodologies, including visual stimulation, auditory simulation, audiovisual combined stimulation, and somatosensory stimulation. Feasible clinical advantages stemming from γ sensory stimulation emanate from alterations in neural function, neural circuitry, and immune signaling pathways

Various stimulation modalities are currently used to induce γ entrainment, including sensory stimulation, optogenetics, transcranial electrical or magnetic stimulation, and deep brain electrical stimulation (Fig. 2) [40].

### Gamma entrainment using sensory stimuli

Various studies on animal models and human diseases have investigated Gamma Entrainment Using Sensory stimuli (GENUS), primarily involving auditory and visual entrainment [20, 32, 49, 50]. The potential clinical benefits of γ sensory stimulation are likely derived from flicker-induced changes in neural function, circuitry, and immune signaling pathways.

### Visual stimulation

The magnitude of visual oscillations is influenced by the frequency, chromaticity, and luminance of the light stimulus [40]. During experiments, participants wear portable opaque eye masks and earplugs while undergoing scalp-EEG recording. They are exposed to flickering light in the γ band to evoke γ oscillations [51, 52]. In animal models, the visual aspect of GENUS involves moving the animals from the holding room to a flicker cage, where light-emitting diodes (LEDs) deliver flickering light at the desired frequency [21].

Brain oscillatory activity is one of the fundamental mechanisms supporting cognitive processes. Present studies indicate that exposure to γ light stimulation leads to functional reorganization across diverse brain regions and modulates functional connectivity within relevant neural networks [53, 54]. EEG results demonstrate γ wave light flicker augments the power of brain oscillations in healthy individuals, emphasizing enhancing activity in the occipital regions bilaterally [55]. Additionally, microglia exhibit a notable affinity for PV + neurons and can restructure perineuronal nets (PNNs), which are crucial for regulating critical period plasticity in the adult cerebral cortex [56, 57]. Evidence shows that exposure to γ wave light flicker reduces PNN coverage in the healthy adult brain and promotes juvenile-like plasticity [56]. In parallel, γ oscillations elicited by light flicker stimulation have been shown to benefit cognitive function and synaptic plasticity in animal models [58, 59]. Prolonged exposure to γ visual flicker drives the reorganization of stress-related neural circuits and enhances hippocampal neuroplasticity in wild-type mice [59]. Visual stimulation with low γ light flicker induces slow γ oscillations in the hippocampal CA1 region, thereby alleviating cognitive impairments in the mouse two-vessel occlusion (2VO) model of cerebral ischemia [58]. Similar investigations in Tau P301S and CK-p25 mice also demonstrated that chronic γ flickering light stimulation enhances functional neuronal connectivity across brain areas, ameliorates neuronal loss, reduces DNA double-strand breaks, offers neuroprotection, and improves spatial memory [21].

Previous studies have shown that flickering light stimulation induces neuronal spiking activity, significantly reducing β-amyloid (Aβ) plaque burden in the visual cortex of 5XFAD mice and facilitating microglial morphological transformation [60]. Furthermore, visual gamma entrainment reduces phosphorylated tau levels in tauopathy mouse models, including P301S and CK-p25, while inducing microglial responses similar to those observed in 5XFAD mice [21]. However, in elderly C57BL/6J mice, γ oscillations induced by visual stimulation did not significantly alter microglial transition to a phagocytic state, microglial quantity, or neuroinflammatory markers [21]. Similarly, in an animal model of ischemic stroke, microglial responses to GENUS appear limited, suggesting that its effects on microglia may depend on disease status or genetic background [13, 58]. As a result, the precise mechanisms and implications of microglial alterations induced by γ wave visual stimulation remain to be determined. In addition, γ stimulation positively modulates neuroimmune biochemical signaling. Exposure to γ flickering lights in wild-type mice upregulates cytokines such as IL-6, and IL-4, enhances microglial phagocytosis, and increases the expression of chemokines, including macrophage colony-stimulating factor and monokines induced by interferon-γ [61]. This neuroimmune activation is mediated by γ-induced phosphorylation of proteins in the nuclear factor κ-light-chain-enhancer of activated B cells (NF-κB) and mitogen-activated protein kinase pathways [61].

### Auditory simulation

Consistent exposure to auditory stimulation has been shown to maintain magnetic field amplitudes in the auditory cortex and induce progressive changes in synaptic efficacy and sensory input, thereby influencing neuronal activity [62]. In animal models, the auditory component of GENUS is administered by exposing animals to tones flickering at the target frequency in a dimly lit, soundproofed room [20]. In humans, auditory stimulation is provided through earphones that emit tones at the specified frequency, while participants wear LED goggles [52, 63]. Previously, auditory cue-triggered neuronal synchronization was discovered and termed Auditory Steady-State Response [64, 65]. It was previously believed that auditory-driven gamma oscillations were restricted to the temporal/auditory cortex [65, 66]. However, recent findings propose that auditory-driven γ oscillations instead encompass the entirety of the cortical mantle [66]. This widespread cortical distribution of auditory-driven γ oscillations is supported by diverse research methods, including whole-head EEG, Magnetoencephalography (MEG), invasive recordings, and electrocorticography (ECoG), which collectively confirm that γ auditory exposure induces γ synchronization across the entire cortical surface [67–70].

Cerebral blood flow (CBF) and vascular changes associated with auditory stimulation-induced γ oscillations have also been investigated [20, 71]. Previous findings suggested that auditory evoked entrainment in healthy humans elicited increased regional cerebral blood flow (rCBF) in the cortex of the posterior aspect of both cerebellar hemispheres [71]. Recent immunostaining studies revealed increased vasodilation and blood vessel diameter in the auditory cortex and CA1 region following chronic auditory stimulation-induced γ entrainment in 5XFAD mice [20]. However, the underlying mechanisms of the interplay between blood flow and γ entrainment remain largely unknown. Interestingly, in a mouse cerebral ischemia model, light flicker failed to increase CBF and blood vessel density [58]. Therefore, further studies are needed to determine whether γ oscillations evoked by auditory entrainment could offer therapeutic benefits for impaired blood supply and vascular damage.

### Audiovisual combined stimulation

Audiovisual stimulation (AVS) is a neurostimulation technique that induces a cerebral response by synchronizing visual and auditory inputs [72]. Specifically, flashing lights are presented to the eyes while pulsed tones are administered to the ears at frequencies associated with brain wave activity, which can be recorded by EEG [73].

According to a recent EEG study, GENUS audiovisual stimulation effectively entrains both cortical sensory regions and deeper brain areas such as the hippocampus, amygdala, insula, and gyrus rectus, noticeably amplifying the power spectral density of frontal and occipital neuron oscillations [74]. In addition, entraining γ oscillations using simultaneous auditory and visual stimulation also influence functional brain connectivity, triggering a change toward normal function [49, 74]. Chronic (8 weeks of daily) audiovisual γ stimulation strengthened functional connectivity between the PCC and PCUN nodes in the DMN of AD patients [49]. PCC-PCUN functional connection strength was positively correlated with cognitive performance [75]. In contrast, another study showed that audiovisual stimulation (3 months daily) did not result in connectivity changes within the DMN but led to a significant increase in mean functional connectivity in the MVN in mild AD patients [74]. However, the authors deemed the observed augmentation in MVN functional connectivity due to the regular use of GENUS light and sound stimulation less probable [74].

In addition to the human study, the beneficial effects of audiovisual combined stimulation have also been demonstrated in a 5XFAD mouse model. Audiovisual stimulation may exert more widespread effects than auditory or visual stimulation. Audiovisual stimulation modality uniquely elicited microglial clustering responses in the auditory cortex, hippocampus, and medial prefrontal cortex and reduced amyloid burden not only in these specific regions but also across the entire neocortex [20]. Furthermore, altered immune factors and cytokines in the cerebrospinal fluid of Alzheimer’s patients following audiovisual γ flicker include downregulation of TGF-α (astrocyte activator), IL-5 (microglial proliferation), MIP-1β (microglial motility), and TWEAK (apoptosis inducer) [49]. Therefore, long-term audiovisual stimulation therapy may attenuate potentially harmful cytokines involved in the activation of microglia and astrocytes. Notedly, TWEAK regulates key immune signaling cascades, including NF‐κB, matrix metalloproteinase, and cellular responses, and results in the disruption of the permeability of the neurovascular unit and blood-brain barrier [76]. Moreover, inhibition of TWEAK may have therapeutic potential in several degenerative diseases [77]. Thus, TWEAK may be a new target for treating neurological diseases through audiovisual stimulation combined with γ oscillations.

### Somatosensory stimulation

The primary modality of γ somatosensory stimulation involves the use of vibrotactile stimuli [78, 79]. The delivery of vibrotactile stimulation was facilitated by an acoustic system that converts γ wave electrical sinusoidal signals to corresponding vertical vibrations [78]. The animal was placed inside a cage on top of a speaker connected to an audio amplifier [78]. In human studies, the participants underwent vibrations while sitting on a vibrating platform chair [79].

External passive γ tactile stimulation induces neural oscillations in the somatosensory cortex [80]. In a clinical study with healthy adult participants, a functional whole-body vibration exercise platform was associated with widespread changes in oxygenated hemoglobin concentration in multiple cortices [81]. Animal studies have corroborated these results following several weeks of daily whole-body γ wave vibrotactile stimulation, which triggered neural activity in the primary somatosensory cortex (SSp) and primary motor cortex (M1), resulting in improved motor performance [78]. Furthermore, after vibration stimulation, the SSp and M1 regions showed decreased phosphorylated tau, synaptic protein loss, DNA damage, and neurodegeneration [78]. Daily vibrotactile stimulation sessions improved anxiety-like behavior, motor performance, and spatial memory in aged rats [82]. In addition, physical exercise combined with γ wave light flickering improves Ca^2+^ homeostasis, reduces reactive oxygen species (ROS), and enhances cognitive performance, mitochondrial function, and neuroplasticity in the 3xTg mouse model [83, 84].

Based on current findings, evidence suggests that inducing γ oscillation stimulation can potentially ameliorate several neuropathologies [85, 86]. However, a significant concern is whether health-related risks occur in the neural circuits of long-term frequent visual flickering γ oscillations [85, 86]. A recent study proposes a novel γ visual entrainment method using Invisible Spectral Flicker (ISF) [87]. Compared to interventions with stroboscopic flicker, ISF induces lower γ amplitude oscillations but exhibits a similar spatial distribution, primarily localized in the posterior electrodes near the visual cortex [87]. Consequently, ISF presents an opportunity for future randomized placebo-controlled clinical trials that substantially reduce the potential for discomfort [87]. In addition, multiple studies have confirmed that GENUS is safe with no serious adverse events and effectively induced γ entrainment with the treatment [49, 74, 88].

### Photobiomodulation

Photobiomodulation (PBM) refers to using low-power light in the visible and near-infrared spectra to induce beneficial biological processes in cells and tissues. Monochromatic wavelengths evoke distinct colors of light on the short-wavelength end of the visible spectrum, including violet (360–400 nm), blue (400–580 nm), and green (560–650 nm) [89].

Current literature suggests that γ rhythm violet optical stimulation through the eyes significantly increases alpha-gamma coupling oscillations, enhancing attention, perception, and memory [54]. Interestingly, exposure to violet light (360–400 nm) has been found to upregulate myopia suppressive gene (EGR-1) expression. EGR-1 is a transcriptional regulator that controls the distribution of methylation sites on brain DNA, which is crucial for neuronal plasticity and memory formation [90, 91]. However, whether specific γ rhythm violet optical stimulation triggers an increase in EGR-1 expression or improves related cognitive functions remains unclear and warrants further investigation. In addition, blue light regulates brain activity patterns more broadly than violet light [89]. Human functional magnetic resonance imaging (fMRI) reveals distinct neural activation patterns in response to γ rhythm blue light exposure through the eyes during a recognition memory task [92]. Furthermore, the γ rhythm visual stimulation-induced neural response exhibits a stronger link to regulating core components within the memory-related network, such as the hippocampus, than exposure to non-flickering natural light [92].

Red-to-infrared light therapy within the 600–1070 nm wavelength range, particularly the near-infrared range, is recognized as a safe and potent therapeutic approach for arresting neuronal degeneration [93]. The application of 1070 nm light stimulation through the scalp and skull at a θ rhythm pulse frequency (10 Hz) activates microglia, leading to morphological changes and enhanced co-localization of microglia with Aβ in APP/PS1 mice, thereby ultimately improving memory ability [28]. Moreover, applying a 1064 nm laser results in significant amplifications of the spectral power strength of electrophysiological oscillations within the alpha (8–13 Hz) and beta (13–30 Hz) bands, observed across a wide range of scalp regions in the human brain [94]. Consequently, employing selective pulse frequencies to manipulate brain oscillations closely linked to specific memory functions may represent a promising strategy to optimize the benefits of light intervention for regulating cognitive function.

### Genetic modifications or optogenetic stimulation

Optogenetic stimulation, a genetic technique that uses genetically engineered cells expressing photosensitive proteins, allows precise activation or inhibition of specific neuronal populations [95]. Moreover, modern optogenetics represents a pivotal milestone in neuroscience, enabling profound insights into the complex orchestration of neural circuitry and behavioral mechanisms while overcoming the limitations of most other methods [96, 97]. Previous research has demonstrated that optogenetic stimulation induces γ rhythms and activates excitatory neurons [98, 99]. For example, constant optogenetic stimulation activates channel rhodopsin 2 (ChR2)-expressing interneurons in the sensory cortex and produces γ band activity in anesthetized cats [27]. In addition, optogenetic stimulation applied to the peri-infarct zone has been shown to effectively restore neuronal activity after stroke in motor and parietal association areas. This also helps attenuate vascular and behavioral dysfunction [29].

Indeed, mounting evidence suggests that optogenetic manipulation of γ oscillations affects neurocircuit function and behavior. For instance, optogenetic activation of γ oscillations in the prefrontal cortex during a goal-directed attentional task improved attentional behavior [100]. Furthermore, optogenetic stimulation of parvalbumin interneurons in the mPFC effectively improved social novelty preference and rescued the social novelty deficit in autism [101]. Similarly, optogenetic stimulation targeting fast-spiking interneurons (FSINs) to induce γ oscillations in the basolateral amygdala has been shown to enhance contextual memory consolidation [102]. The crucial role of the Dlx5/6 gene in the development of GABAergic interneurons provides further evidence supporting the impact of γ stimulation on circuit function and behavioral flexibility, as demonstrated in experiments with Dlx5/6+/− mice [69, 103]. In these mice, the abnormality of FSINs occurs during adolescence, coinciding with the onset of cognitive inflexibility and compromised task-evoked γ oscillations [69]. However, optogenetic induction of γ oscillations in the PFC effectively restored cognitive flexibility in Dlx5/6+/− mice, enabling them to perform the task consistently over an extended duration [69].

Although optogenetic stimulation has demonstrated neuroprotective effects, the mechanisms by which γ entrainment in various brain regions affects Aβ deposition remain unclear. For example, optogenetic manipulation of γ oscillations in CA1 neurons has been linked to reduced Aβ levels in both 5XFAD and APP/PS1 mouse models [60]. Conversely, a separate study found that optogenetic stimulation of PV + neurons in the basal forebrain of 5XFAD mice increased amyloid burden in the frontal cortical region [104]. Similarly, optogenetic stimulation of medial septal PV + neurons rescue the amplitude of hippocampal low-frequency γ oscillations and enhances spatial memory performance despite significant plaque deposition [2]. Hence, it is hypothesized that divergent stimulation modalities elicit distinct molecular and cellular responses. These responses may involve different action mechanisms, potentially entraining γ oscillations within a complex neurocircuit that spans multiple brain regions [13]. Along with meticulously designed clinical trials, further investigations are warranted to elucidate these limitations, ascertaining whether induction methods have potentially positive or harmful impacts on pathology.

### Transcranial electrical stimulation

Transcranial electrical stimulation (TES) is a non-invasive technique that delivers controlled electric fields to the scalp to directly modulate cerebral activity through low voltage constant or alternating currents [105]. TES encompasses a range of methodologies, including transcranial direct current stimulation (tDCS), transcranial random noise stimulation (tRNS), transcranial alternating temporal interference (tTIS), and transcranial alternating current stimulation (tACS) [106–108]. Specifically, tDCS modulates cortical areas by delivering low-intensity direct current, which induces bidirectional, polarity-dependent changes in spontaneous neuronal activity [109]. Meanwhile, tDCS exhibits remarkable tolerability in humans, allowing for a comprehensive assessment of neuropsychological, physiological, and motor effects in clinical research [109]. In comparison, tRNS delivers low-intensity, randomly alternating biphasic current directly to the scalp and elucidates the modulatory effects of cortical excitability on motor learning and perceptual processing [110]. Furthermore, tTIS is a non-invasive method for achieving focal and steerable deep brain stimulation, which involves applying high-frequency alternating currents at distinct scalp sites [106]. Finally, tACS entails the administration of sinusoidal alternating electric currents with specific frequencies in pre-defined cerebral regions across the scalp to primarily impact endogenous oscillatory activity in the brain [111]. Additionally, tACS is intended to modulate cerebral function and influence cognitive processes by entraining brain oscillations and enhancing neural communication [112]. Although tDCS and tRNS effectively modulate cortical excitability and plasticity, tACS uniquely targets frequency-specific modulation of oscillatory dynamics. Meanwhile, current literature insinuates that tACS applied at γ frequencies effectively modulates various cerebral functions [19, 113]. Thus, this section will investigate the theoretical and practical applications of tACS.

In a randomized, double-blind, sham-controlled crossover pilot study, the impact of transcranial alternating current stimulation at γ frequency (γ-tACS) or sham tACS was meticulously explored in patients with mild cognitive impairment [19]. Notably, the active γ-tACS intervention involves a solitary 60-minute treatment session, precisely targeting the Pz region (an area overlying the medial parietal cortex and the precuneus), which is known to play a pivotal role in the episodic memory network [114]. Compared to the sham exposure, γ-tACS yielded significant improvements in memory performance and reinstated intracortical connectivity measures of cholinergic neurotransmission [19]. A subsequent study examined the effects of γ-tACS on episodic memory and cholinergic transmission in patients with Alzheimer’s [113]. The 60-minute treatment targeted the precuneus with either γ-tACS or a sham intervention. Results showed a significant correlation between improvements in episodic memory and indirect measures of cholinergic neurotransmission following active γ-tACS [113]. Pre- and post-EEG assessments revealed increased γ-power activity in posterior brain regions, indicating the localized impact of γ-tACS on the precuneus, posterior parietal cortex, and cognitive function [113].

Recent investigations have unveiled a causal nexus between γ oscillations and preparatory and execution stages of movement [115]. Targeting the application of γ-tACS within the M1 enhances the velocity and acceleration of visually triggered movements, contrasting with the negligible impact of beta-tACS or sham stimulation [115]. These improvements induced by γ-tACS are significantly associated with the altered blood oxygenation level-dependent activity localized to the stimulated M1 region and task-specific modulation of neural activity in the distant dorsomedial prefrontal cortex [115]. Additionally, γ-tACS is related to the motor performance of tasks requiring motor control, like visuomotor performance [116]. Applying 70 Hz tACS over the M1 and cerebellar cortex significantly improved performance on an isometric force task involving visuomotor control of the right index finger, particularly in healthy individuals with suboptimal baseline motor performance [116]. Similarly, stimulation at a high γ frequency (80 Hz) enhances motor performance during a visuomotor coordination task [117]. Thus, the involvement of high-frequency motor cortex γ oscillations in complex visuomotor tasks involves abrupt adjustments to motor planning and execution [117]. In addition, γ oscillations in cortical motor areas reflect synaptic activity and contribute to plasticity [118]. Previous studies indicate that γ-tACS combined with intermittent θ burst stimulation (iTBS) induces LTP-like plasticity in the M1 of healthy individuals [119]. Clinical research also shows that γ entrainment (70 Hz) via tACS improves motor impairment in PD patients and modulates GABAA activity in M1 [43, 118]. Specifically, γ-tACS reverses LTD-like effects and enhances LTP-like plasticity by inhibiting GABAergic interneurons in M1 [43]. Thus, γ-tACS can potentially reverse LTD-like plasticity in the human M1. In addition, working memory is a complex cognitive function involved in temporary information storage and manipulation, making it a target for neurorehabilitation [120].

Several studies suggest that tDCS also modulates γ activity. During a visual task, administration of tDCS to the occipital cortices results in augmented local γ oscillation amplitude [121]. Remarkably, tDCS also unravels network-level ramifications, characterized by heightened γ oscillations in the prefrontal cortex, parietal cortex, and various visual attention regions [121]. Similarly, anodal tDCS applied to the dorsolateral prefrontal cortex significantly increases γ power and improves working memory performance in patients with SCZ [122]. In addition, vagus nerve stimulation (VNS), another γ-band stimulation methodology, involves the modulation of the vagus nerve through electrical impulses [123]. The vagus nerve traverses the neck, forming a neural pathway that links peripheral organs with lower regions of the brain [123]. Vagal nerve branches intricately innervate anatomical structures associated with human memory processing within complex neuronal networks [123]. γ entrainment using transcutaneous auricular vagus nerve stimulation (γ-taVNS) efficiently reduces hippocampal amyloid load in APP/PS1 mice [124]. Furthermore, γ-taVNS elicits microglial phagocytosis and regulates microglial pyroptosis by effectively suppressing the P2 × 7R/NLRP3/caspase-1 pathway in the hippocampus [124]. Additionally, γ-taVNS exerts inhibitory effects on the hippocampal NF-κB pathway, increasing neuroprotection, spatial memory, and learning [124].

### Transcranial magnetic stimulation

Transcranial Magnetic Stimulation (TMS) is a non-invasive medical procedure that uses magnetic fields to stimulate nerve cells in the brain [105]. It involves placing a coil near the scalp, generating magnetic pulses that pass through the skull and penetrate targeted brain regions [105]. Recent studies show that periodic electromagnetic force engendered through rhythmic TMS modulates brain function [125]. Notably, rhythmic TMS fosters the regulation of brain oscillations by perturbing and realigning ongoing oscillatory activities [125]. The most commonly used TMS method is repetitive transcranial magnetic stimulation (rTMS), capable of inducing time-varying magnetic fields within the cerebral cortex [126]. These evoked magnetic fields generate action potentials within specific neurons of targeted brain regions by eliciting electric currents in rhythmic patterns [126]. Recently, γ-band rTMS treatment amplified power in the γ frequency band within the left temporoparietal cortex, improving cognitive and executive functions by facilitating local, long-range, and dynamic connectivity within the brain regions, promoting information flow and integration [127].

Interestingly, all patients maintained favorable health status, without any documented unwanted reactions during therapy, indicating the safety and feasibility of γ-rTMS intervention [127]. Enhancing γ oscillatory activity through rTMS applied to the dorsolateral prefrontal cortex has emerged as a promising cognitive enhancement strategy for neuropsychiatric disorders characterized by cognitive impairments [128]. Compelling evidence highlights the ability of rTMS to target the dorsolateral prefrontal cortex and to induce normalizing excessive gamma oscillations in individuals with schizophrenia and ASD [129, 130]. Furthermore, rTMS elicits plasticity-like changes in cortical function and behavior, improving language function in healthy individuals and various aspects of memory in patients with severe depression [128].

## Gamma brain stimulation for Alzheimer’s disease

AD is one of the most prevalent neurodegenerative diseases, pathologically characterized by excessive extracellular Aβ accumulation and intracellular tau hyperphosphorylation [131, 132]. Although numerous studies have been conducted over the past decades to treat AD by targeting Aβ and abnormal tau, nearly all clinical trials targeting Aβ and tau hyperphosphorylation have failed [133]. Therefore, the Aβ and tau hypotheses have been questioned in recent years [134].

The pathological buildup of amyloid-beta oligomers (Aβo) disrupts the synchronized generation of action potentials in pyramidal cells and disturbs the balance of excitatory and inhibitory processes within the hippocampal network [135]. This disruption results in impaired hippocampal theta-gamma phase-amplitude coupling and compromised long-term potentiation (LTP), which are crucial for memory encoding and cognitive function [136, 137]. PV + and somatostatin-positive (SST) interneurons represent the prominent subtypes of interneurons in the hippocampus, playing a pivotal role in θ -nested γ oscillogenesis and LTP induction [137, 138]. Specifically, PV + interneurons selectively modulate γ oscillations, while SST + interneurons modulate θ oscillations [60, 139]. Dysfunction in SST + and PV + interneurons contributes to impairments in θ and γ oscillations observed in an AβO-injected mouse model of AD [140]. Thus, Aβo causes synapse-specific dysfunction in PV + and SST + interneurons, likely contributing to impaired hippocampal γ oscillations and synaptic plasticity in AD [137]. In AD mouse models, the regulatory capacity of inhibitory interneurons to maintain oscillatory rhythms and network synchrony crucial for cognitive function is compromised [48, 141]. Notably, the dysfunction of Nav1.1-dependent interneurons is functionally significant in the pathogenesis of AD-associated cognitive impairments [141]. Efforts to restore the normal levels of Nav1.1 facilitate the enhancement of γ-oscillatory activities, mitigate excessive network synchrony, and alleviate cognitive decline in hAPP mice [142].

**Fig. 3:**
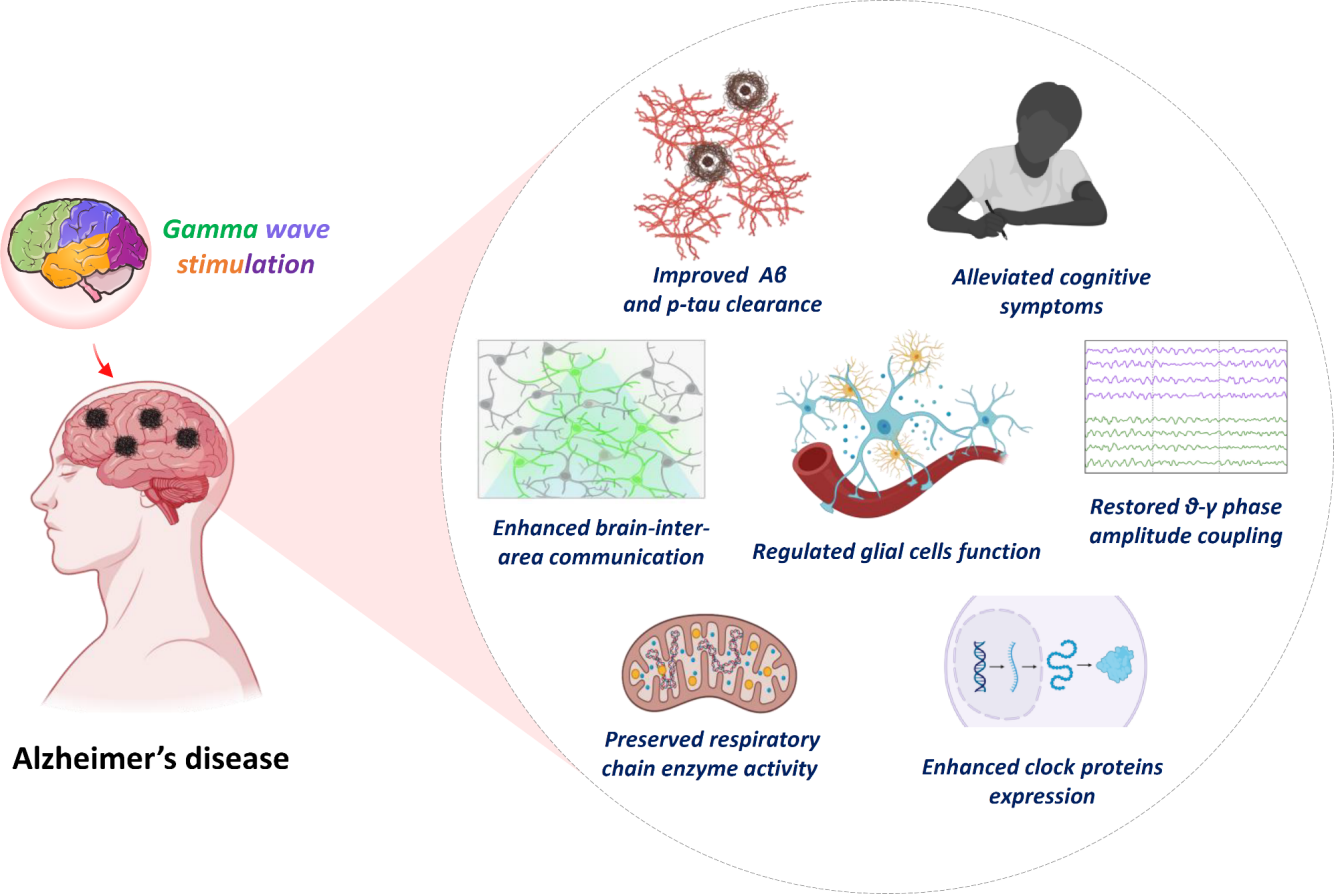
The beneficial effect of gamma stimulation in AD. Gamma stimulation confers various benefits on AD, including enhancing brain-inter-area communication, improving Aβ and p-tau clearance, regulating glial cell function, preserving respiratory chain enzyme activity, alleviating cognitive symptoms, and enhancing clock protein expression

The alteration of neuronal network activity may predate the onset of AD, potentially occurring before the deposition of Aβ and leading to changes within the hippocampal network [143, 144]. In the early stages of the disease, abnormal slow γ oscillations are observed during hippocampal sharp wave ripples (SWRs) in AD mouse models [60]. The gradual decline in slow γ activity initiated by interneurons during SWRs significantly contributes to apoE4-mediated learning and memory impairments [14]. SWRs originate in hippocampus and are triggered by synchronized activation of CA3 pyramidal neurons, leading to high-frequency oscillations in the local field potential recorded from the CA1 region [145]. During SWRs, slow gamma oscillations are elevated, and the increased gamma synchrony between CA3 and CA1 is associated with more coordinated neuronal firing [146]. Restoring slow γ oscillations during SWRs is critical for modulating memory retrieval. In addition, accumulation of the 1N3R isoform of tau within astrocytic processes in the dentate gyrus of AD patients triggered mitochondrial relocation and impaired motility in hilus astrocytes, diminishing γ oscillations and PV-expressing neurons, resulting in spatial memory impairments [147]. On the other hand, before the deposition of Aβ plaques and the onset of cognitive impairments, individuals with AD exhibit olfactory dysfunction characterized by an inability to perceive and identify odors [148]. With advancing age, Aβ aggregation induces the dysfunction of reciprocal dendrodendritic synapses between granule cells and mitral cells, consequently leading to aberrantly enhanced γ oscillations and olfactory impairment [149]. Thus, considering γ oscillations as potential biomarkers for preclinical AD is rational (Fig. 3) [150].

Mounting evidence suggests γ-band oscillations (especially 40 Hz) are critical for multiple sensory and cognitive processes [3, 151, 152]. Previous studies confirm that cognitive activity induces 40 Hz event-related potential in humans [153]. Similarly, researchers discovered that compared with healthy people, Alzheimer’s patients had reduced 40 Hz brainwaves in the cortical component [154]. In the 5XFAD mouse model, exposure to flicker stimulation at various frequencies (including 8 Hz, 40 Hz, 80 Hz, random stimulation, and no stimulation) revealed that 40 Hz (1 h per day, 7 days) flicker significantly reduced Aβ plaque burden in the visual cortex [60]. Similarly, the application of 40 Hz (1 h/d day, 7 days) γ visual stimulation enhanced gamma power among several brain areas, including the visual cortex, hippocampus, prefrontal cortex, and somatosensory cortex, leading to improvements in associated cognitive symptoms and neurodegeneration [21, 60]. Interestingly, random stimulation resulted in increased Aβ levels, suggesting that different types of visual stimuli may elicit distinct effects [60]. Similarly, auditory or audiovisual stimulation at 40 Hz (2 h/day, 14 days) improves cognitive performance and mitigates neuropathological alterations in apoE4 knock-in mice while also reducing neuronal apoptosis and enhancing cholinergic transmission in the hippocampus [155]. Another study used transcranial-focused ultrasound pulsed at 40 Hz, decreasing Aβ plaque deposition in 5XFAD mouse models [156]. Notably, 40 Hz flickering also improves mitochondrial function. In the Aβ1–42 toxicity condition (as an AD model), utilizing a 40 Hz flickering white LED has been shown to improve the structural and functional integrity of ion channels, particularly mitoBKCa channel, and promote mitochondrial respiratory chain enzyme activity, specifically complex I and IV [157, 158]. Furthermore, evidence suggests that 40 Hz light simulation enhances mitochondrial membrane potential (ΔΨm) and mitigates ROS production in mouse models of AD [157].

Sleep and circadian dysfunction commonly occur in AD patients, partly contributing to the progression of neurodegeneration [159, 160]. A recent study demonstrates that 40 Hz (1 h/day, 30 days) light simulation ameliorates circadian rhythm disturbance in the APP/PS1 AD mouse model, restoring the hypothalamus electrophysiological changes, reducing Aβ deposition in the hypothalamus, and enhancing rhythmic expression of clock proteins, including BMAL1, CLOCK, and PER2 [22, 161]. Specifically, pretreatment with 40 Hz (1 h/day, 30 days) flickering light alleviated disrupted circadian rhythms, improved the ratio of nighttime to total activity, and corrected fragmented rest periods in AD mice [22]. Furthermore, after 30 days of 40 Hz flickering light treatment, no adverse effects on body weight, blood glucose levels, heart rate, or biological rhythms were observed in the mice [22]. Overall, 40 Hz entrainment exhibited positive outcomes across various AD pathology animal models (including 5XFAD, Tau P301S, APP/PSI, and CK-p25), indicating that the effects are not model-specific [162, 163].

However, despite these promising results, it is important to note that other studies could not replicate these outcomes. Specifically, a recent study showed that both acute (10-minute baseline followed by one-hour stimulation) and chronic (one hour per day for seven days) 40 Hz visual flickering failed to entrain deeper brain structures in APP/PS1 and 5XFAD models [31]. Only a small fraction of neurons responded to light stimulation, with no detectable effects on intrinsic γ oscillations [31]. Furthermore, the results revealed no overt reliably reduced Aβ load in the neocortex or hippocampus or alteration in microglial morphology within the experimental animals [31]. Optogenetic stimulation was employed to selectively activate medial septal PV neurons at different γ-band frequencies in the cortex of J20-APP animal models [2, 164]. Although 40 Hz stimulation successfully restored hippocampal slow γ oscillation amplitude and phase-amplitude coupling, effectively rescuing spatial memory deficits, Aβ plaque deposition persisted [2]. Likewise, 40 Hz (1 h/day, 4 weeks) tACS did not substantially impact Aβ burden but did reduce p-Tau levels within the specific temporal lobe area in AD patients [165]. Given the constraints of small sample sizes, varying treatment protocols, and diverse evaluation criteria in clinical trials, large-scale studies are needed to establish a robust therapeutic phenotype for γ entrainment in AD pathology.

## Gamma brain stimulation for Parkinson’s disease

PD is characterized by dopaminergic neuron depletion, α-synuclein (α-Syn) misfolding and aggregation, mitochondrial dysfunction, neuroinflammation, and oxidative stress [166]. PD is clinically manifested by motor symptoms such as resting tremor, bradykinesia, rigidity, and postural instability, as well as non-motor symptoms like REM sleep disorder, anosmia, cognitive impairment, and depression [167]. Pharmacological interventions, such as dopamine replacement therapy, remain the predominant treatment modality. However, these treatments show diminishing efficacy over time, potentially leading to motor complications [166]. Increasing investigations into non-invasive neurostimulation and neuromodulation techniques have emerged as alternative strategies to address PD pathology.

**Fig. 4:**
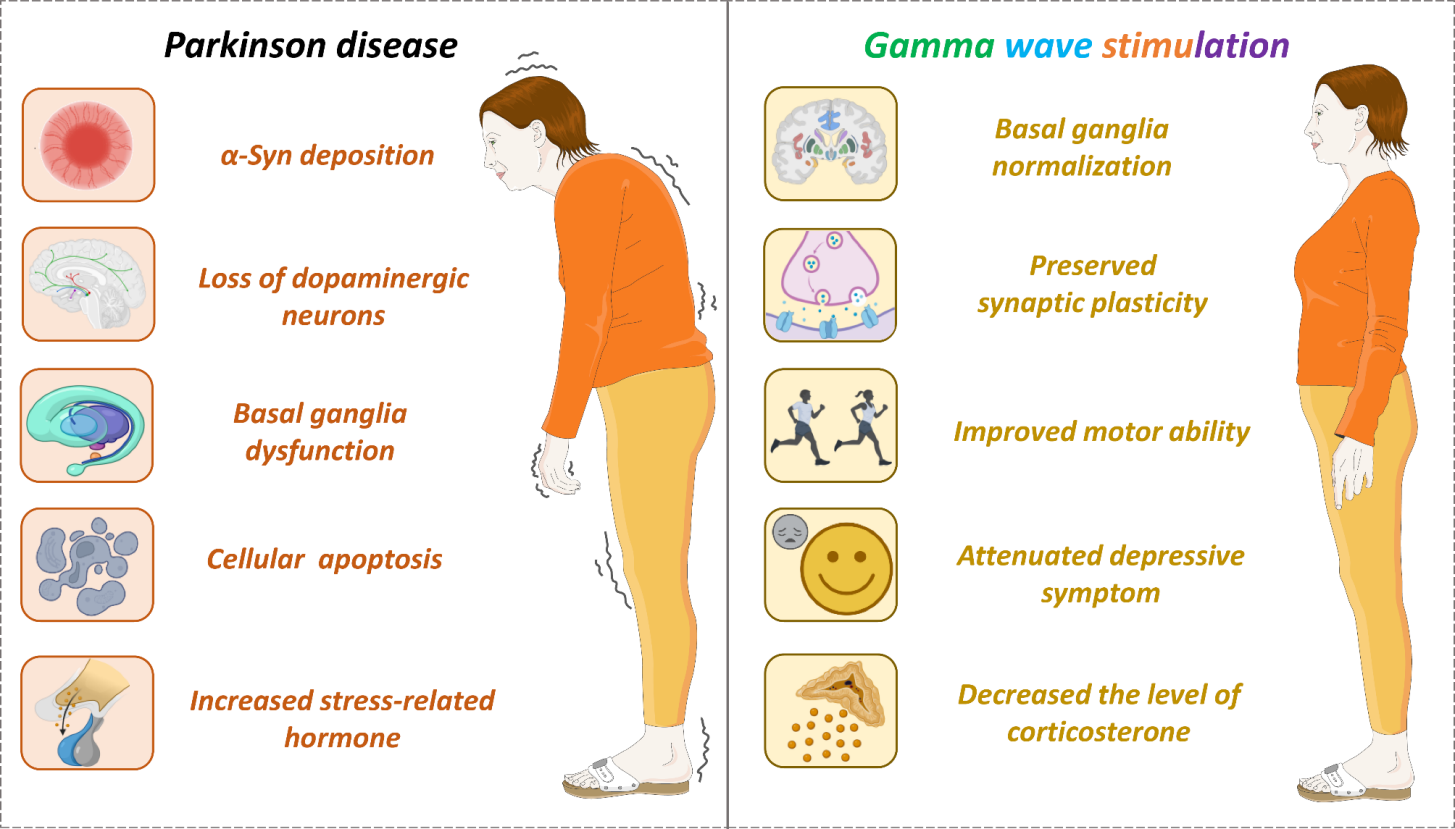
The beneficial effect of gamma stimulation in PD. Gamma stimulation improves basal ganglia normalization, preserves synaptic plasticity, alleviates the increase in stress-related hormones, and improves behavioral changes

Under the decreased burst rate of the hypodopaminergic state, a deficiency in regulating subcortical γ signaling may contribute to the pathomechanism underlying bradykinesia in PD (Fig. 4) [168]. Furthermore, dopamine loss disrupts the basal ganglia, a brain structure responsible for regulating motor function [168]. Enhancing gamma oscillations restores synaptic plasticity in the cortical motor regions [43, 169]. Recent neurophysiological studies show reduced long-term potentiation (LTP)-like plasticity in M1 and diminished γ oscillations within the basal ganglia-thalamo-cortical network in PD patients [43, 170]. The specific γ oscillatory activity ranging from 60 to 90 Hz is relevant to the motor network and exhibits correlated changes with movement execution [171]. The combination of tACS delivered over the cortical motor areas at 70 Hz and intermittent θ burst stimulation demonstrates that driving γ oscillations restores LTP-like plasticity in patients with PD [43]. Furthermore, a double-blind, randomized controlled trial suggests that 40 Hz vibration (25 min/day, 12 weeks) through psychoacoustic therapy improves tremor, rigidity, bradykinesia, posture, and gait in PD patients [172].

Additionally, GENUS has the potential to facilitate aberrant protein clearance and treat non-motor symptoms in PD animal models. Prolonged multisensory 40 Hz (2 h/day, 1 month) stimulation effectively reduces p-α-Syn deposition in the cortex and striatum [173]. However, 40 Hz (2 h/day, 1 month) audiovisual stimulation ameliorates neuromuscular strength, spatial working memory, and depressive behaviors in A53T PD mice [173]. Thus, γ stimulation has the potential to modify PD progression.

## Gamma brain stimulation for stroke

Stroke is a prominent cause of mortality and functional impairment that results from a transient or lasting decrease in cerebral perfusion [174]. After a stroke, neurons may undergo persistent depolarization, worsened by impaired interneuron function, which typically inhibits adjacent excitatory neurons [29]. During a stroke, rapid and extensive deterioration occurs within the neuronal structure and function, with limited restoration during reperfusion [175]. Moreover, the delicate balance between excitatory and inhibitory processes is disrupted, leading to reduced cerebral activity that impedes the dynamic reorganization of functions after the stroke [175]. However, the persistence of γ oscillations in the affected hemisphere is positively correlated with rehabilitation progress in stroke patients, suggesting that γ oscillations are integral to the post-stroke recovery process [44]. Hence, γ oscillation synchronization is strongly associated with clinical outcomes in stroke rehabilitation survivors [44].

**Fig. 5:**
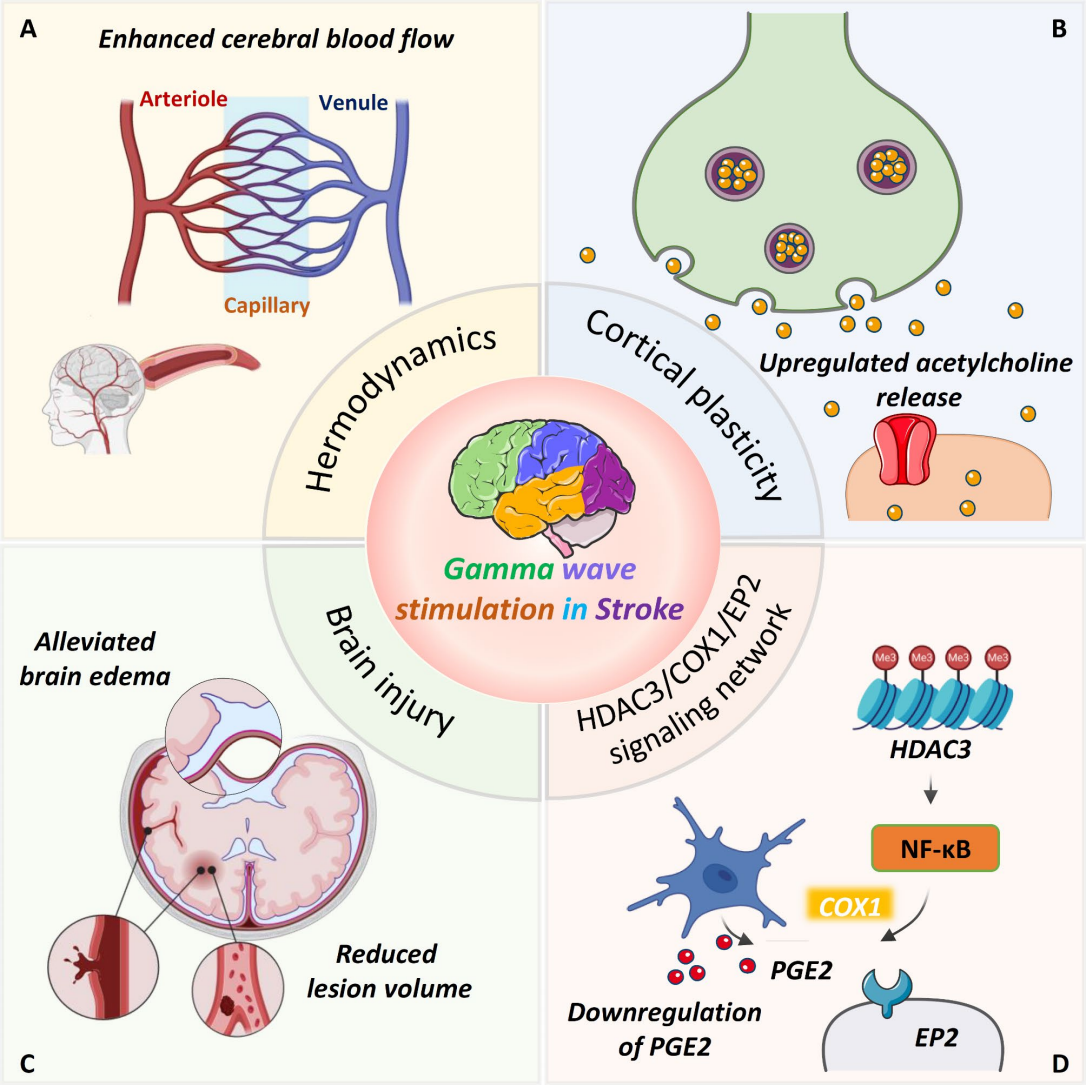
The beneficial effect of gamma stimulation in stroke. Gamma stimulation confers various benefits on stroke, including preserving synaptic plasticity, alleviating lesion volume, and maintaining cerebral blood flow. Additionally, γ stimulation downregulates the HDAC3/COX1/EP2 network and alleviates deficits in behavioral changes

Recent findings suggest modulating cortical oscillatory dynamics during the acute phase may offer neuroprotection against stroke (Fig. 5) [29]. In the acute phase following stroke, optogenetic stimulation of fast-spiking interneurons at 40 Hz in the lesioned hemisphere activates inhibitory interneurons in the M1, reducing the incidence of spreading depolarizations [29]. Subsequently, activation of interneurons at 40 Hz alleviates brain edema and lesion volume, enhances cerebral blood flow, and improves behavioral outcomes of post-stroke mice [29]. In addition, the cholinergic neurons of the basal forebrain exert influence over an array of functions, including cortical plasticity, attention, and sensorimotor behavior [175]. Research indicates that acetylcholine (ACh) regulates cortical plasticity during the acute phase after stroke, playing a key role in recovery and compensation [176]. Thus, ACh innervation in the neocortex is thought to play a significant role in post-stroke recovery [176]. Optogenetic stimulation of the nucleus basalis during the post-stroke period increases ACh release, improving functional recovery and motor behavior in the photothrombotic stroke mouse model [175].

Deficits in specific hippocampal oscillation frequencies are closely linked to cognitive dysfunction in the ischemic brain. Previous studies suggest that reduced cross-frequency coupling between θ and γ rhythms in hippocampal local field potentials is associated with impaired short- and long-term potentiation in the 2VO rat model [177]. Additionally, a persistent reduction in low γ oscillations has been identified in an anesthetized mouse model of unilateral hippocampal ischemia [178]. Visual stimulation at low γ frequency (30–50 Hz, 1 h/day, 14 days) restores phase-amplitude coupling with θ oscillations and rescues cognitive dysfunction in the 2VO mouse model [58]. Mechanistically, γ frequency sensory entrainment enhances synaptic plasticity via RGS12-regulated N-type CaV2.2 voltage-gated calcium channels (N-VGCC) [58].

Most evidence indicates that post-stroke phobic anxiety is widely prevalent, impeding the rehabilitation of patients and disrupting their usual activities [179]. Post-stroke anxiety is mediated by the up-regulation of histone deacetylase 3 (HDAC3) in activated microglia residing within the ischemic cortex, which facilitates the deacetylation process, subsequently leading to the nuclear translocation of p65 and activation of the NF-κB pathway [13, 180]. The activation of the NF-κB pathway further upregulates downstream target genes involved in prostaglandin synthesis, including cyclooxygenase-1 (COX1) and prostaglandin E2 (PGE_2_) [13, 180]. Subsequently, the interaction between PGE_2_ and EP2 receptors in the amygdala enhances anxiety and depression susceptibility to stress following ischemic stroke [13, 180]. Importantly, it is worth noting that γ flicker stimulation has shown efficacy in inhibiting the activation of cortical microglia, down-regulating the HDAC3/COX1/EP2 signaling network, and alleviating anxiety-like behaviors in the photothrombotic stroke mouse model [180].

## Gamma brain stimulation for schizophrenia

SCZ presents as a prominent psychotic disorder with manifestations of positive symptoms (hallucinations and delusions), negative symptoms (avolition and anhedonia), and impairments in the prefrontal cortex-dependent cognitive domains, encompassing attention, cognitive flexibility, working memory, and social cognition [181]. Cognitive dysfunction is a fundamental characteristic of SCZ [182]. Cognitive impairments persist continuously throughout the illness, which is strongly correlated with long-term functional prognosis and frequently preceding the onset of overt psychosis [182, 183]. Regrettably, existing antipsychotic treatments exhibit only marginal efficacy in addressing cognitive symptoms [183]. Dysbindin-1, a protein containing a coiled-coil domain, exhibits reduced levels within the cerebral cortex of individuals afflicted with SCZ [184]. Gamma-frequency neuronal firing facilitates the translocation of dysbindin-1 into mitochondria, where it interacts with Drp1 and related receptors, inducing the formation of oligomeric Drp1 complexes that promote mitochondrial fission [184, 185]. As a result, *Drp1* deficiency may diminish mitochondrial fission and disrupt γ oscillations in mouse models [185]. However, the augmentation of mitochondrial fission using a light-responsive mitochondrial fission system offers a potential solution to restore the integrity of the γ rhythm [185].

Disrupted GABAergic signaling and diminished activity of NMDA receptors are pivotal components in the pathophysiology of SCZ, disrupting the balance between excitation and inhibition in cortical and subcortical networks leading to abnormal neural oscillations [181]. While performing tasks requiring cognitive control, individuals with SCZ exhibit observable deviations in the PFC γ activity and concomitant impairments in PV + neuron functionality [45, 103]. Therefore, the pathological mechanisms that influence PV + neurons detrimentally affect γ oscillations and the synchronization of cortical neural activity, contributing to the cognitive dysfunction observed in SCZ [45].

**Fig. 6:**
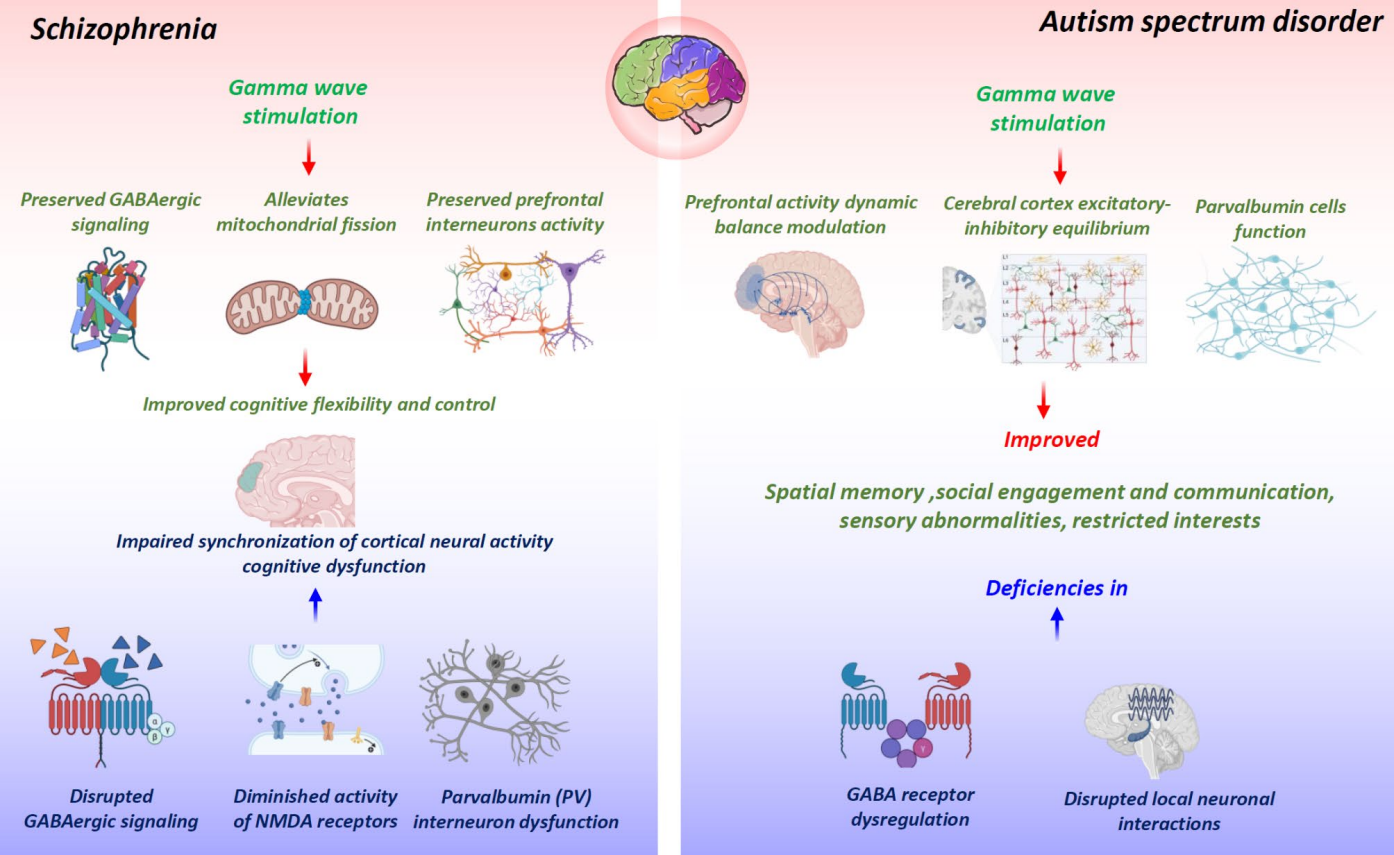
The beneficial effect of gamma stimulation in SCZ and ASD. Gamma stimulation confers various benefits on SCZ and ASD, including preserving GABAergic signaling, alleviating mitochondrial fission, enhancing prefrontal interneuron activity, improving cognitive flexibility and control, maintaining prefrontal activity dynamic balance modulation, preserving cerebral cortex excitatory-inhibitory equilibrium and parvalbumin cell function, and alleviating behavioral changes

Previous findings demonstrate that optogenetic stimulation effectively overcomes the inherent cognitive impairment in the SCZ mouse model, resulting in long-lasting cognitive flexibility improvements (Fig. 6) [69]. Remarkably, cognitive benefits from interneuron stimulation occur only when γ-frequency stimulation is applied at 40–60 Hz, not with stimulation protocols combining higher and lower frequencies [69]. Hence, γ-frequency activity originating from prefrontal interneurons is crucial in cognitive functions central to SCZ [69]. Nevertheless, future investigations must explore the mechanisms by which interneuron-driven γ oscillations facilitate cognitive enhancement. Additionally, patients diagnosed with SCZ exhibit impairments in high-frequency γ (≥ 60 Hz) oscillations, particularly during visual processing [186], suggesting that gamma entrainment techniques could offer a promising therapeutic intervention for these visual processing abnormalities.

## Gamma brain stimulation for autism spectrum disorder

ASD is a multifactorial condition influenced by genetic and environmental factors, leading to persistent deficiencies in social engagement and communication, sensory abnormalities, restricted interests, and repetitive behaviors [187]. ASD often co-occurs with disorders such as anxiety, depression, attention-deficit/hyperactivity disorder (ADHD), and obsessive-compulsive disorder (OCD), contributing to significant impairments in activities of daily living (ADLs) in both pediatric and adult populations [46]. Research shows that ASD is characterized by disrupted neuronal interactions within local networks, leading to aberrant γ-frequency brainwave activity patterns [188]. In patients with ASD, reduced interneuron numbers and dysregulated GABA receptor subunit expression reflect an imbalance between excitatory and inhibitory signaling, primarily mediated by the GABAergic pathway [38]. Similarly, several studies in ASD patients have found reduced spontaneous γ activity in frontal, temporal, and right-lateral regions, reduced left-hemispheric MEG steady-state γ responses, reduced task-related γ power, and reduced long- and short-range γ connectivity [46]. Deviant patterns of evoked and induced γ oscillations elicited by sensory tasks have likewise been documented in individuals with ASD within the visual domain [189] and the auditory domain [190]. Given the various body of evidence, we posit that abnormal γ frequency activity should be regarded as an integral component within the expansive pathophysiological construct of ASD (Fig. 6) [126, 191].

Many scholarly investigations within the medical domain substantiate a robust association linking the functionality of PV cells, γ oscillations, and impairments in social cognition [126]. Disruptions in inhibitory feedback, mediated by fast-spiking interneurons, lead to imbalances in excitation and inhibition within prefrontal circuits among young individuals, resulting in reduced coherence in evoked γ frequency synchronization [192]. These extensive alterations at structural and functional levels culminate in attenuated cognitive capabilities and impaired social proficiency [192]. Consequently, dynamic modulation of prefrontal activity during the early stages of neurodevelopment plays a pivotal role in governing the cognitive competence of adults, potentially exerting a critical impact on the manifestation of cognitive symptoms in neuropsychiatric disorders [192]. Manipulation of γ oscillations, particularly within the dorsolateral prefrontal cortex, correlates with enhancements in cognitive abilities, corrections of the excitatory-inhibitory balance within the cerebral cortex, and improvement of social deficits [126, 129]. Recent literature suggests that TMS therapy over the dorsolateral prefrontal cortex in ASD patients normalizes γ band irregularities, enhances cognitive functioning, and improves socio-behavioral impairments [187].

Dysfunction in synaptic neurotransmission may underlie intricate modifications in neural circuits, contributing to behavioral phenotypes in ASD [193, 194]. Deletion of the autism-associated *Cntnap2* gene disrupts the density of PV + interneurons within the hippocampus, leading to imbalances in inhibitory neurotransmission in the perisomatic region [193]. Reduction in PV + interneuron density leads to decreased inhibition of CA1 pyramidal cells, resulting in deficits in spatial discrimination and alterations in frequency-dependent circuit dynamics in the hippocampus, such as disrupted γ oscillations, sharp-wave ripples, and theta-gamma modulation [193]. Current evidence suggests that the real-time modulation of the excitation-inhibition balance in the prefrontal cortex of Cntnap2-null mutant mice effectively alleviates social behavior deficits reminiscent of autism phenotypes [194]. Currently, γ entrainment of medial septal PV + interneurons restores aberrant low-frequency γ oscillation amplitudes and theta-gamma phase-amplitude coupling within the hippocampus, ameliorating spatial memory deficits [2]. Consequently, harnessing the potential of γ entrainment to enhance hippocampal circuit dynamics in ASD might yield similar benefits and warrants further investigation.

## Discussion and future directions

Gamma oscillations are essential for sensory processing, memory consolidation, and cognitive function and are attenuated in neurodegenerative diseases and other brain disorders. Various techniques for brain stimulation have been shown to induce gamma oscillations. Importantly, synchronized light and sound stimulation at 40 Hz effectively induces corresponding brain activity at the same frequency [13, 195]. Overall, 40 Hz GENUS is associated with reduced neuroinflammation, enhanced synaptic transmission, and increased expression of genes related to synaptic plasticity. These effects lead to improvements in cognitive function [21, 23, 24, 58, 83, 196, 197]. Furthermore, 40 Hz GENUS leads to an increase in the expression of cytokines in microglia, normalization of circadian rhythms, and a reduction in Aβ plaque burden [61, 198, 199]. Additionally, other studies have demonstrated that multisensory 40 Hz stimulation enhances the glymphatic clearance rate of Aβ [200]. Despite these promising findings, some studies have failed to replicate these results. Another study has demonstrated that 40 Hz optogenetic stimulation effectively modulated spatial memory, while plaque loads were not altered [2]. Additionally, several studies have reported failures to replicate the natural γ oscillations, Aβ reduction, and microglial activation observed with 40 Hz GENUS [31]. The complex pathological changes associated with AD may lead to variability in the accuracy and effectiveness of the 40 Hz stimulation protocol, depending on different stages of the pathogenesis of AD [63]. Thus, possible reasons for the discrepancies above lie in the variations in stimulation modalities and assay time relative to stimulation. To better understand the discrepancies between these results and previously reported findings, it is essential to investigate specific parameters (such as optimal color, intensity, and frequency) that can effectively induce gamma entrainment. Research on flicker light stimulation for γ wave entrainment in humans indicates that pure white light at a brightness level of 400 cd/m² and a flicker frequency of 34–38 Hz may represent the most effective strategy for achieving γ entrainment [86]. In addition to stimulation parameters, another potential contributing factor to the discrepancies observed between studies may be individual variances among animals or patients and their specific responses to the stimulation. Animals or patients exhibit slight differences in their processing of visual sensory stimuli between dark and light cycles, which can result in distinct behavioral responses [55]. For example, a study that applied 40 Hz visual stimulation during the dark phase observed an increase in anxiety-like behaviors in 5XFAD mice, potentially due to differences in brain states and neuroregulatory systems associated with circadian rhythms [31]. Furthermore, it is crucial to investigate whether the presence of aversive behaviors could diminish the gamma entrainment effect and impede the clearance of amyloid proteins. Other studies reporting positive outcomes performed light stimulation during non-aversive phases [60, 74]. Therefore, establishing appropriate control groups to examine the influence of environmental factors on the effectiveness of GENUS interventions is imperative. In fact, non-invasive acoustic stimulation experiments demonstrate that, in both animals and humans, the application of slow oscillatory sound stimuli during sleep can enhance γ oscillations, potentially improving circadian mechanisms and sleep quality [22, 198].

The neuroprotective effects of induced gamma activity, particularly through 40 Hz GENUS, are promising. However, several questions remain about the underlying molecular pathways and the roles of different cell types, such as neurons and glial cells. Future investigations are essential to clarify the cellular mechanisms that regulate brain oscillations, thereby enhancing understanding of the neuroprotective mechanisms that mitigate disease progression. While the pronounced neuroprotective effects of 40 Hz GENUS in various neurodegenerative disease models are noteworthy, several unresolved questions remain and warrant further exploration. Notably, most existing studies concentrate on the early stages of pathological changes, leaving unanswered whether 40 Hz GENUS can reverse substantial neuronal loss once damage has occurred. In addition, different diseases or disorders may have specific frequency characteristics [186, 201]. For instance, the 60–90 Hz frequency range is associated with bradykinesia [202], while γ wave anomalies in the range of 40 Hz to 100 Hz are related to the spectrum of SCZ [203]. Consequently, future research should recognize that variations in gamma frequency across different diseases necessitate disease-specific applications of sensory entrainment. Additionally, it is crucial to assess whether acute or chronic interventions with GENUS result in greater improvements in brain function. While GENUS has demonstrated safety and feasibility in humans and positive outcomes in various animal studies, previous research has been limited by small sample sizes [49, 74, 198, 204]. Therefore, large-scale clinical trials are indispensable for rigorously assessing the efficacy of GENUS in improving disease outcomes. Additionally, determining how long the neuroprotective effects of GENUS last after cessation is crucial, as this information could inform long-term treatment strategies for sustained therapeutic benefit.

## Conclusions

Natural gamma and 40 Hz sensory-induced steady-state oscillations likely engage distinct neurobiological mechanisms. Thus, elucidating the mechanisms underlying spontaneous, sensory-evoked, and optogenetically induced gamma entrainment could provide critical insights into the nature of brain oscillations. In summary, the 40 Hz GENUS, with its ability to modulate higher-order emotional and cognitive processing via multiple pathways, exerts pervasive effects on the brain, potentially mitigating pathological states. Thus, further investigation into the neurobiological mechanisms behind induced gamma activity could lead to novel therapeutic strategies for treating neurological disorders.

## Data Availability

Not applicable.

## Abbreviations

AD: Alzheimer’s disease
EEG: Electroencephalography
GABA: γ-aminobutyric acid
PD: Parkinson’s disease
SCZ: Schizophrenia
ASD: Autism spectrum disorder
GENUS: Gamma ENtrainment Using Sensory stimuli
Aβ: Amyloid-beta
NF-κB: Nuclear factor κ-light-chain-enhancer of activated B cells
LTD: Long-term depression
PNN: Perineuronal nets
ROS: Reactive oxygen species
MEG: Magnetoencephalography
CBF: Cerebral blood flow
PCC: Posterior cingulate cortex
DMN: Default mode network
ISF: Invisible Spectral Flicker
PBM: Photobiomodulation
FSINs: Fast-spiking interneurons
TES: Transcranial electrical stimulation
tDCS: Transcranial direct current stimulation
tRNS: Transcranial random noise stimulation
tTIS: Transcranial alternating temporal interference
tACS: Transcranial alternating current stimulation
TMS: Transcranial Magnetic Stimulation
SWR: Sharp wave-ripple
α-Syn: α-synuclein
LTP: Long-term potentiation
